# Child Life under Queen Victoria

**Published:** 1897-02-27

**Authors:** 


### CHILD LIFE UNDER QUEEN VICTORIA.

### III.—THE FIRST FACTORY ACTS.

THE first Factory Act was introduced in 1802. It may, therefore, be regarded as outside our purview, all the more that it proved absolutely futile. But it is worth recording that it was a master manufacturer, Sir Robert Peel, who, in face of the determined opposition of his fellows, thus tried to protect the factory apprentices. There was as yet no protection for free labour. When, however, the introduction of steam-power caused factories to be built in populous districts, and children were no longer apprenticed, Sir Robert Peel brought in another Bill, in 1815, to protect this "free labour," as it was curiously called. Unfortunately, in his desire to conciliate opposition, Sir Robert yielded one point after another, till his Bill was so mutilated as to be comparatively useless. Still, it brought forward the principle that children, whether under the charge of their parents or not, should be protected from the effects of unlimited competition, that *laisser faire* system which the economists of that day held up as almost a divine revelation. Manufacturers protested in all earnestness that if they were compelled to limit their hours of work they would have to close their mills, not recognising the difference between the powers of steam and steel and those of a human being. Therefore young children were to trot twenty-five to thirty miles a day in attendance on the spinning-mule, until, at the time when they should have been at their full strength, they were worn-out cripples. Even the most hardened economists were forced to admit that to use up the strength of a life-time within the first twenty years of life was false economy after all.

In 1819 another law was passed forbidding the employment of children under nine years of age, and limiting the hours of work for all persons under sixteen to twelve per day, less one and a half for meals. But as none of these Acts contained any provision for inspection, the alleviations they provided, such as they were, were almost completely ignored. In 1825 Sir John Hobhouse brought in a Bill which reduced the work on Saturdays to nine hours—the first step to the Saturday half-holiday. Various difficulties in the interpretation of this Act were settled by others that were passed during the next few years. In the reign of William IV. Sir John Hobhouse brought forward a new Bill, and with this the factory agitation may be said to have reached its climax. The names of Sadler, Fielden, and Oastler became prominent. The Bill was mutilated out of knowledge. All that was attained was that young persons under the age of twenty-one should not be employed at night work. The modest demand of Sir J. Hobhouse for a working day of eleven and a-half hours was rejected. The workmen responded by demanding a day of ten hours, and eight on Saturday—a working week of fifty-eight hours instead of sixty-nine. The question of legislative interference with the hours of labour had been raised, and could not now be ignored.

In 1832 Mr. Sadler brought forward his Bill, but, as if to help the obstructives, the Reform Bill was passed; Newark, for which borough Sadler sat in the House of Commons, was disfranchised; and the champion of the factory workers failed to obtain another seat. Looking around for another Parliamentary supporter, they were led to entrust their cause to Lord Ashley, known to this generation as the seventh Earl of Shaftesbury. Personally no better choice could have been made, and Lord Ashley nobly justified the confidence placed in him. But his position as a member of the land-owning class, the heir to an ancient title, caused the enemies of the cause he championed to taunt him with ignoring the evils that lay at his own door.

When Lord Ashley announced his intention of re-introducing Mr. Sadler's Bill, the Government announced its intention of appointing a Royal Commission to visit the factory districts and conduct inquiries on the spot. This Commission was believed, by those who supported as well as by those who opposed it, to have the intention of all previous Commissions, namely, to postpone legislation. But in two months the Commissioners presented their first report, which distinctly stated that in view of "permanent deterioration of the physical constitution, the production of disease wholly irremediable, and the partial or entire exclusion (by reason of excessive fatigue) from the means of obtaining adequate education, and acquiring useful habits, or of profiting by those means when afforded," they were of opinion that a case is made out for the interference of the Legislature on behalf of children employed in factories. On this deliverance was founded Lord Althorp's Act.

This Act forbade the employment for more than nine hours a day of any child under eleven years of age, or within thirty months after the passing of the Act any child under thirteen. The point, however, which made this law more efficient in action than its predecessors was the appointment of factory inspectors. Hitherto there had been no efficient inquiry as to whether such provisions as existed were carried out; now employers found themselves face to face with fines, and even imprisonment, if they neglected the law. And yet there were still so many evasions of the law, and the Government seemed so little anxious to make their own Act sufficiently operative, that in 1838 Lord Ashley had to protest in the House of Commons against its inefficiency. Already the Government had brought in a measure intended to repeal the protection given to children over twelve years of age. This meant, as Lord Ashley said, "to legalise the slavery of some forty thousand children, for the most part females." So intense was the opposition to this Bill that the Government found it wise to drop it. In 1839 a Factory Amendment Act was passed, but Lord Ashley's demand for the inclusion of workers in silk mills in its protection formed an excuse for abandoning it. A committee was, however, formed for inquiring into the working of the existing Act. This committee made various suggestions for preventing infringements of the Act, which were incorporated in the next Factory Act, that of 1844. This Bill included women in the protection hitherto given only to children. The hours for children's work were now limited to six daily, and a certain amount of education was rendered compulsory. Provision was also made for fencing dangerous machinery. But it was not till 1847 that the final battle of factory legislation was won. The Ten Hours Bill was passed, and, after forty-five years of struggle, our legislature accepted the protection of labour as one of its duties.

